# Hybrid-gate MoS_2_ 2T0C DRAM for low-power multi-bit storage with high linearity

**DOI:** 10.1093/nsr/nwaf555

**Published:** 2025-12-05

**Authors:** Zhejia Zhang, Saifei Gou, Yufei Song, Xiangqi Dong, Yuxuan Zhu, Zhengjie Sun, Mingrui Ao, Qicheng Sun, Jinshu Zhang, Yan Hu, Yuchen Tian, Haojie Chen, Xinliu He, Jieya Shang, Qihao Chen, Yang Liu, Yin Xia, Chen Yang, Hao Meng, Mingyuan Liu, Huihui Li, Yin Wang, Peng Zhou, Wenzhong Bao

**Affiliations:** State Key Laboratory of Integrated Chip and Systems, School of Microelectronics, Fudan University, Shanghai 200433, China; State Key Laboratory of Integrated Chip and Systems, School of Microelectronics, Fudan University, Shanghai 200433, China; State Key Laboratory of Integrated Chip and Systems, School of Microelectronics, Fudan University, Shanghai 200433, China; State Key Laboratory of Integrated Chip and Systems, School of Microelectronics, Fudan University, Shanghai 200433, China; State Key Laboratory of Integrated Chip and Systems, School of Microelectronics, Fudan University, Shanghai 200433, China; State Key Laboratory of Integrated Chip and Systems, School of Microelectronics, Fudan University, Shanghai 200433, China; State Key Laboratory of Integrated Chip and Systems, School of Microelectronics, Fudan University, Shanghai 200433, China; State Key Laboratory of Integrated Chip and Systems, School of Microelectronics, Fudan University, Shanghai 200433, China; State Key Laboratory of Integrated Chip and Systems, School of Microelectronics, Fudan University, Shanghai 200433, China; State Key Laboratory of Integrated Chip and Systems, School of Microelectronics, Fudan University, Shanghai 200433, China; State Key Laboratory of Integrated Chip and Systems, School of Microelectronics, Fudan University, Shanghai 200433, China; State Key Laboratory of Integrated Chip and Systems, School of Microelectronics, Fudan University, Shanghai 200433, China; State Key Laboratory of Integrated Chip and Systems, School of Microelectronics, Fudan University, Shanghai 200433, China; State Key Laboratory of Integrated Chip and Systems, School of Microelectronics, Fudan University, Shanghai 200433, China; State Key Laboratory of Integrated Chip and Systems, School of Microelectronics, Fudan University, Shanghai 200433, China; State Key Laboratory of Integrated Chip and Systems, School of Microelectronics, Fudan University, Shanghai 200433, China; Shaoxin Laboratory, Shaoxing 312000, China; Changxin Memory Technologies, Hefei 230601, China; Changxin Memory Technologies, Hefei 230601, China; Changxin Memory Technologies, Hefei 230601, China; Changxin Memory Technologies, Hefei 230601, China; Shaoxin Laboratory, Shaoxing 312000, China; State Key Laboratory of Integrated Chip and Systems, School of Microelectronics, Fudan University, Shanghai 200433, China; Shaoxin Laboratory, Shaoxing 312000, China; State Key Laboratory of Integrated Chip and Systems, School of Microelectronics, Fudan University, Shanghai 200433, China; Shaoxin Laboratory, Shaoxing 312000, China; Shanghai AtomIC Technology, Shanghai 201318, China

**Keywords:** MoS_2_, 2T0C DRAM, low-power consumption, multi-bit storage, high linearity

## Abstract

With the increasing demand for high-performance computing, 2T0C DRAM has been extensively studied for its high integration density, low power consumption and non-destructive readout. Two-dimensional (2D) semiconductors, with ultra-low leakage current, improve the retention characteristics but face limitations in conventional 2D-based 2T0C cells: the subthreshold operation of positive-threshold transistors at low write voltages reduces read current, introduces nonlinearity, and degrades robustness, and thus requires higher write voltages and increased power consumption. To address this, we propose a hybrid-gate MoS_2_ 2T0C DRAM, where a low-leakage Au-gate transistor serves as the write node and a depletion-mode Al-gate transistor functions as the readout node. The device achieves >100 s retention time and reduces the minimum write voltage to 0.2 V, enabling distinguishable 3-bit storage. Furthermore, a 32 × 32 MoS_2_ 2T0C DRAM circuit demonstrates image storage and readout capabilities with <5% bit error rate after 600 s, highlighting its potential for future high-density, low-power memory applications.

## Teaser

This work develops a novel low-power memory cell using hybrid-gate MoS2 transistors, enabling multi-bit storage and reliable image retention with high linearity.

## INTRODUCTION

With the rapid advancement of information technology and smart devices, the demand for high-performance computing accelerator chips is surging [1,2]. However, memory access latency and high power consumption have emerged as primary bottlenecks limiting computational capability [3]. Consequently, the development of high density, high performance, low power memory devices and computing-in-memory (CIM) architectures is an urgent challenge. Embedded memory compatible with logic processes, such as embedded DRAM (eDRAM), has been widely adopted in high performance computing processors [4–13]. The most common eDRAM architectures include the 1T1C (one transistor, one capacitor) and 2T0C (two transistors, no capacitor) structures. Unlike the 1T1C structure [14–16], the 2T0C architecture utilizes the gate capacitance of the read transistor (T_read_) to store information, thus eliminating the need for an additional capacitor [5–10]. This structure further reduces the area and process complexity of the DRAM cell. Furthermore, the 2T0C structure separates the write and read paths, enabling non-destructive readout and avoiding frequent refresh operations, thereby significantly lowering power consumption [17]. However, the performance of the 2T0C structure depends critically on precise threshold voltage (*V*_TH_) control and leakage current suppression of the transistors. To achieve extended retention time at near 0 V hold voltage (*V*_hold_), the write transistor (*T*_write_) must exhibit ultra-low off-state leakage current [4,18], and its *V*_TH_ needs to be controlled within a relatively positive range. However, maintaining an appropriate *V*_TH_ is critical, as an excessively high *V*_TH_ value would substantially reduce the sensing window during the write operation [19]. For the *T*_read_, a depletion mode design is essential to compensate for the contraction of the sensing window during the reading process. Employing the same fabrication process for both the *T*_write_ and *T*_read_ transistors compromises the device’s ability to detect small voltage signals, thereby significantly increasing write power consumption. In amorphous oxide semiconductors (AOSs), ITO has been employed as the *T*_read_ material to address this issue [10,20]. Nevertheless, heterogeneous on-chip integration poses substantial challenges in terms of material growth and device scaling processes. Consequently, the development of homogeneous materials and novel architectures of 2T0C DRAM has emerged as a crucial research priority.

In recent years, two-dimensional (2D) materials, particularly transition metal dichalcogenides (TMDs) such as molybdenum disulfide (MoS_2_), have attracted considerable interest in memory device research due to their exceptional electronic properties, ultrathin thickness, and excellent electrostatic control [21–23]. Leveraging its atomic-layer thinness and intrinsic high carrier mobility, monolayer MoS_2_ demonstrates exceptional capability in suppressing short-channel effects and minimizing leakage current, while simultaneously enabling high gate capacitance density and robust electrical stability [24,25]. These advantages make MoS_2_ an ideal candidate for next-generation high-density, high-performance DRAM devices [26]. Previous research has shown that MoS_2_-based 2T0C DRAMs exhibit high retention characteristics and low leakage current, with heterogeneous integration in large scale integrated circuit fabrication [18,27].

To address the above challenges, this study proposes a novel 2T0C DRAM structure with a high sensing window and low power consumption. The structure is based on MoS_2_ semiconductors grown by chemical vapor deposition (CVD) and fabricates a hybrid-gate using hetero-process techniques. For the first time, an Al gate depletion mode *T*_read_ with a negative *V*_TH_ is introduced to mitigate the effective shrinkage of the sensing margin caused by the *V*_TH_ of *T*_read_. Additionally, the relatively large drive current improves read accuracy and linearity. In addition, the *T*_write_ employs a high-work-function Au gate to achieve a *V*_TH_ close to 0 V, resulting in a lower leakage off-state at the same *V*_hold_ and longer retention time of MoS_2_-based 2T0C DRAMs. Based on these hetero-processes, we fabricated a hybrid-gate 2T0C DRAM with a long retention time exceeding 100 s at room temperature, demonstrating quasi–non-volatile characteristics. The minimum voltage of the write bit line (*V*_WBL_) has been reduced by a factor of 30 to 0.2 V, while enabling distinguishable 3-bit data storage. Furthermore, a 32 × 32 2T0C DRAM circuit has been implemented to demonstrate image storage and retrieval functionality. The Al-gate process integrated in the hybrid-gate strategy significantly enhances the linearity of the write/read operations. The image bit error rate (BER) remains below 5% even after 600 s, ensuring high-fidelity data retrieval. This study establishes a scalable and energy-efficient 2T0C DRAM architecture with enhanced retention, multi-bit storage, and low-voltage operation, demonstrating a significant step forward in embedded memory design. In addition, the successful integration of wafer-scale CVD-grown MoS_2_ further highlights its immense potential for next-generation high-performance memory devices.

## RESULTS AND DISCUSSION

### 2T0C DRAM cell structure and electrical test results

**Figure 1.**
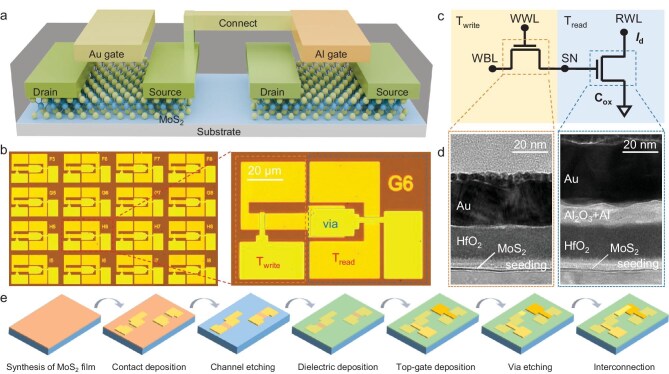
The architecture of the Au/Al gate 2T0C device. (a) Schematic of 2T0C DRAM device structure based on single-layer MoS_2_. (b) Optical microscope image of 2T0C DRAM cell and array. (c) Schematic circuit diagram of the 2T0C cell. (d) Transmission electron microscope (TEM) image of the channel area cross-section of the Au gate and Al gate transistors. (e) Manufacturing process flow of a 2T0C DRAM cell.

Figure 1a illustrates the device structure proposed in this study, which consists of a 2T0C DRAM device composed of monolayer MoS_2_ top-gate transistors with hybrid-gate fabrication processes (Au/Al gate 2T0C). The dimensions of the two transistors are both 30 μm (W) × 10 μm (L). The optical microscope images of the device and array are shown in Fig. 1b. As shown in the circuit schematic of the 2T0C device in Fig. 1c, the source of the *T*_write_ is connected to the gate of the *T*_read_, with the gate capacitance of the *T*_read_ serving as the storage node. The switching state of the *T*_write_ is controlled via the write word line (WWL) and WBL, allowing for the writing, erasing, and retention of charge information at the storage node. During the reading process, a constant source-drain voltage (RWL) is applied to the *T*_read_, and the current is sampled. The storage node voltage (*V*_SN_) is obtained by fitting the drain current, enabling non-destructive reading of the information.

**Figure 2.**
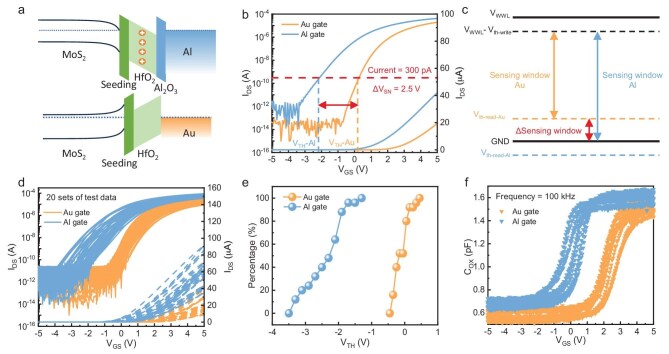
Energy band structure and performance of Au gate/Al gate MoS_2_ transistors. (a) Schematic diagram of the energy bands of Au/Al gate transistors. (b) Comparison of the transfer characteristic curves of Au/Al gate transistors. (c) Schematic of the sensing margin of Au/Al gate 2T0C and Au/Au gate 2T0C. (d) Transfer characteristic homogeneity of Au/Al gate transistors. (e) Threshold voltage probability distributions for Au/Al gate transistors. (f) Capacitance-voltage (*C−V*) results for Au/Al gate transistors.

This study proposes a hybrid-gate strategy and introduces the Al gate process for fabricating the *T*_read_ for the first time. The cross-sectional TEM image of the channel region of the Al gate transistor compared to the Au gate transistor is shown in Fig. 1d. Different metal gates significantly affect the *V*_TH_ of MoS_2_ field-effect transistors (FETs). Figure 2a presents the energy band diagrams of Au gate and Al gate transistors. Due to the higher work function of Au (5.1 eV) compared to Al (4.08 eV), the lower work function of the Al gate induces electron accumulation in the channel under zero gate bias, while the higher work function of the Au gate depletes the channel of charge [28]. Furthermore, at the Al/HfO_2_ interface, the Al layer absorbs oxygen atoms from HfO_2_, oxidizing into a dense Al_2_O_3_ layer, which generates more oxygen vacancies (V_O_) in the high-k HfO_2_ dielectric layer. These V_O_ induce an electrostatic dipole effect, bending the energy bands at the MoS_2_ interface. As a result, compared to the Au gate transistor, the Al gate transistor shows stronger electron doping characteristics in the transfer characteristics curve, with a notable shift of the *V*_TH_ in the negative direction [29].

Figure 2b presents the comparison of the transfer characteristics between the Au gate and Al gate transistors (the orange curve represents the Au gate, and the blue curve represents the Al gate), in which the drain-source current is set to 1 V. The *V*_TH_ is defined as the gate-source voltage (*V*_GS_) at which the source-drain current reaches 100 pA*(W/L). It is evident that the *V*_TH_ of the Au gate transistor is significantly higher than that of the Al gate transistor, with a *V*_TH_ difference of ∼2.5 V. In the 2T0C structure, when reading through *T*_read_, there is typically a read-out sensing margin reduction contributed by the *V*_TH_ of *T*_read_. For the Al gate transistor, since the *V*_TH_ is notably lower than 0 V, even when the storage node voltage (*V*_SN_) is low, *T*_read_ can still maintain a significant drain current. Therefore, within the voltage range between GND and *V*_WWL_—*V*_TH-w__r__i__te_, the Al gate transistor is capable of effective readout. In contrast, the *V*_TH_ of the Au gate transistor is >0 V. When *V*_SN_ approaches 0 V, the *T*_read_ operates in deep subthreshold, resulting in negligible read-out current on the read bit line (RBL) (Fig. 2c). Thus, within the voltage range from GND to *V*_TH-Au_, the Au gate transistor operates in the subthreshold region, which is highly sensitive to noise and process instability, and its limited driving current restricts both read speed and amplitude, thereby hindering effective information retrieval. Therefore, the implementation of Al gate transistors significantly mitigates the sensing window reduction caused by the *V*_TH_ of *T*_read_ in the 2T0C structure, thereby enhancing read-out performance and stability.

To further evaluate device consistency, we investigate the performance uniformity of MoS_2_ transistors fabricated with Au and Al gates. Figure 2d presents the transfer characteristic curves of 20 Au gate transistors and 20 Al gate transistors, in which the drain-source current is still set to 1 V. It is clearly evident that the *V*_TH_ values of the Au gate transistors are concentrated around 0 V to 0.5 V, while the *V*_TH_ of the Al gate transistors are distributed between −3 V and −2 V, with the on-state current of the Al gate transistors approximately twice that of the Au gate transistors. These data indicate that the Al gate process enables a stable and reliable doping effect, with good uniformity and yield. Additionally, cycling endurance tests were performed (see detailed data in Sections S9 and S18), in which it is observed that the device maintains excellent performance even after experiencing 10^9^ programming/erasing cycles, demonstrating outstanding operational stability. We further extracted the *V*_TH_ distribution of MoS_2_ transistors with two different top-gate processes, as shown in Fig. 2e, demonstrating that the introduction of the Al gate process effectively achieved a negative *V*_TH_ shift of ∼2.5 V. The capacitance-voltage (*C*-*V*) test results of 10 sets of Au gate and Al gate transistors at a frequency of 100 kHz are shown in Fig. 2f. The *V*_TH_ was determined by the maximum slope point of the *C*−*V* curve, and the *V*_TH_ difference (∼2.5 V) obtained from the *C*-*V* statistical plot corresponds to the difference in *V*_TH_ extracted from the transfer characteristic curve, which further confirms our theoretical analysis.

The incorporation of readily oxidizable aluminum in the gate structure may raise legitimate concerns regarding long-term device stability. To address this critical issue, we compared the electrical characteristics of Al gate transistors measured over a 100-day interval. As demonstrated in Fig. S12, the transfer characteristics remain essentially unchanged, with key performance parameters showing negligible degradation. These results provide compelling evidence for the exceptional long-term stability of our aluminum-gate architecture.

### Storage performance improvement for 2T0C DRAM

**Figure 3.**
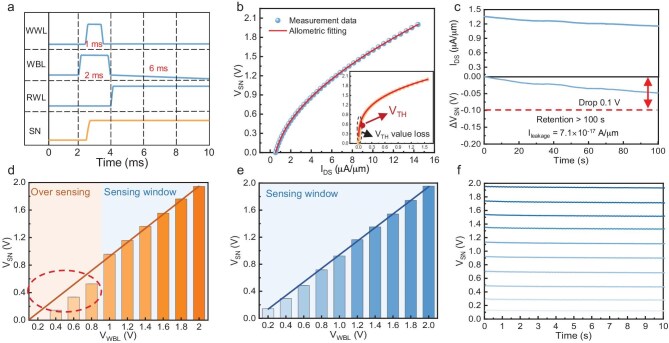
Memory characteristics of 2T0C cells. (a) Signal waveform plots of write and read operations to the 2T0C cell in one cycle. (b) Allometric polynomial fitting of the obtained *V*_SN_-*I*_DS_ plot. (c) Retention characteristics of the hybrid-gate 2T0C cell. *V*_WBL_ and *V*_SN_ correspondence of the (d) Au/Au gate 2T0C and (e) hybrid-gate 2T0C. (f) Retention characteristics of the hybrid-gate 2T0C cell at different *V*_SN_.

Next, we tested the storage performance of the 2T0C memory cells based on hybrid-gate processes. Figure 3a shows the signal waveform for the write and read operations of the 2T0C cell within one cycle. During the write operation, a 3 V pulse with a duration of 1 ms is applied to *V*_GS_ (WWL) and a 2 V pulse with a duration of 2 ms is applied to *V*_DS_ (WBL). The *V*_GS_ pulse is delayed by 1 ms compared to the *V*_DS_ pulse. Under these conditions, *T*_write_ is fully turned on, and the *V*_SN_ is written with a voltage value of 2 V. After the pulses, the V_WWL_ is set to −2 V, the *V*_WBL_ is set to 0 V, and the leakage path of *T*_write_ is cut off. During the read operation, a 2 V V_DS_ pulse is applied to *T*_read_, with *V*_SN_ acting as the *V*_GS_ of *T*_read_, modulating the *I*_DS_ of *T*_read_. This means that the *V*_SN_ can be inferred by measuring the *I*_DS_ of *T*_read_. We obtained the relationship between *V*_SN_ and I_DS_ through polynomial allometric fitting of *T*_read_’s transfer characteristic curve, as shown in Fig. 3b. The inset of Fig. 3b shows the fitting result when using an Au gate transistor as the *T*_read_. Comparison shows that when using an Al gate transistor as the *T*_read_, the fitted *V*-*I* curve exhibits a better linear relationship, allowing for accurate current measurement and information reading even at very small *V*_SN_ values. However, when using an Au gate transistor as the *T*_read_, for *V*_SN_ ≤ 0.5 V, the read-out operation imposes high demands on the external read-out circuit due to the limited read-out current. On the other hand, the fitted *V*−*I* curve has poor linearity, and it is difficult to effectively distinguish *V*_SN_ within this range, leading to potential read errors. Based on the above analysis, significant accuracy loss in the 2T0C cell makes it challenging to achieve effective read-out under *V*_SN_ conditions below the *V*_TH_. The introduction of the Al gate structure can effectively mitigate the contraction of the sensing window issue.

Figure 3c shows the retention characteristics of the proposed Au/Al gate 2T0C structure. The upper graph illustrates the I_DS_ measured after continuous reading for 100 s, while the lower graph depicts the change in *V*_SN_ (Δ*V*_SN_) obtained by fitting the *I*−*V* curve. Typically, the retention time of a 2T0C DRAM cell is defined as the time it takes for *V*_SN_ to drop by 0.1 V [6–8,10,30]. As observed from the graph, after 100 s, the decrease in *V*_SN_ remains below 0.05 V, indicating that the proposed 2T0C structure exhibits an ultralong retention time of over 100 s, with the leakage current calculated to be 7 × 10^−17^ A/µm. These results demonstrate that the Au/Al gate 2T0C DRAM possesses excellent storage characteristics and ultra-low leakage, making it suitable for the storage requirements of the vast majority of application scenarios. Additionally, multiple sets of retention data and endurance performance were tested (see detailed data in Sections S6, 10, and S14), confirming the consistency of the leakage current level.

Figure 3d and e further substantiates the effectiveness of Al gate transistors as *T*_read_ in mitigating the contraction of the sensing window, based on experimental measurements. During the write operation, the *V*_WBL_ was incremented from 0 to 2 V in 0.2 V steps, and the storage node voltage under various conditions was measured. As shown in Fig. 3d, the shrinkage of the sensing window was caused when the Au gate transistor was used as the *T*_read_ at the *V*_WBL_ below 1 V. This is manifested by a nonlinear relationship between the *V*_WBL_ and the *V*_SN_, with significant distortion observed in the *V*_SN_ values derived from the *I*_DS_ of *T*_read_, presenting a major challenge for high-precision multi-bit storage applications. However, when an Al gate transistor was used as the *T*_read_, this issue was notably improved. The test results for the Al gate transistor show that a good linear relationship between *V*_SN_ and *V*_WBL_ is maintained, and even when the *V*_WBL_ is as low as 0.2 V, the *V*_SN_ derived from current fitting can effectively reflect the voltage state of the storage node. This indicates that the application of Al gate transistors in 2T0C DRAM can better accommodate the read-out requirements at low voltages, effectively avoiding the accuracy issues caused by shrinkage of the sensing window.

The write power consumption was calculated using the minimum *V*_WBL_ required to maintain linear input-readout characteristics (1 V for Au/Au gate 2T0C, 0.2 V for Au/Al gate 2T0C), and the implementation of a hybrid-gate process reduced the write power consumption of the 2T0C cell by more than one order of magnitude (see detailed calculation in Section S7).

Based on the significant advantages of the Au/Al gates 2T0C device in terms of high linearity and low power consumption, we further tested its storage volume under different *V*_SN_, as shown in Fig. 3f. The results indicate that, regardless of the initial storage voltage of the *V*_SN_, no significant decrease in *V*_SN_ was observed within a 10 s storage time. This demonstrates that the hybrid-gate 2T0C not only enhances read accuracy but also ensures stable charge retention characteristics, providing reliable support for multi-bit storage implementation.

**Figure 4.**
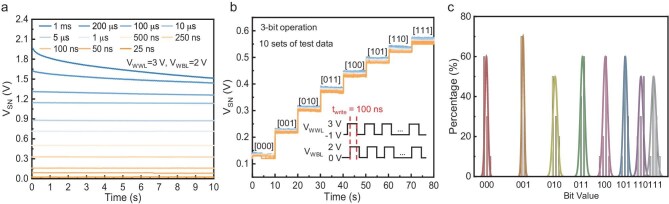
Write speed and 3-bit memory of Au/Al gate 2T0C device. (a) *V*_SN_ of the Au/Al gate 2T0C at different write times. (b) 3-Bit memory of the Au/Al gate 2T0C. (c) Distribution statistics of 3-bit values in 10 tests.

Write speed is also an important evaluation criterion for DRAM. As the write time is reduced from 1 ms to 25 ns, the *V*_SN_ shows a downward trend (Fig. 4a). Based on the above write speed characteristics, we set the write time of pulse to 100 ns and consecutively applied 8 write pulses to obtain 8 significantly distinguishable storage node voltage values, thereby achieving 3-bit storage. To ensure the reliability and uniformity of the test results, Fig. 4b presents 10 sets of test data, which exhibit a high degree of overlap and distinguishability. Furthermore, the distribution of 3-bit values in 10 tests was statistically analyzed, and it can be observed that all 8 values achieved good differentiation (Fig. 4c). These results indicate that the proposed 2T0C structure has potential for multi-bit storage applications, offering promising prospects for the future manufacturing of high-density DRAM.

### Image storage and readout

**Figure 5.**
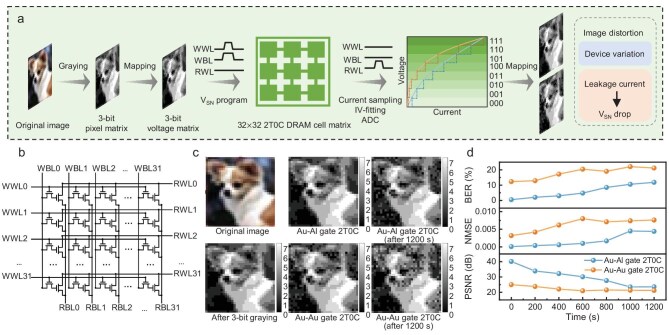
Image storage and comparison of readout quality. (a) Schematic of the operation of an Au/Al gate 2T0C DRAM for image storage and readout. (b) Schematic of the 32 × 32 2T0C DRAM array circuits. (c) Information of the original image, the greyscale image and the readout of the image after 0 s and 1200 s of storage using the Au/Al gate 2T0C DRAM and the Au/Au gate 2T0C DRAM, respectively (images copyrighted from Stanford Dogs Dataset). (d) Comparison of BER, NMSE and PSNR of images read from the two DRAM circuits.

Based on the low write power, ultra-long retention time, multi-bit storage, and high linearity readout characteristics of the Au/Al hybrid-gate 2T0C device, we fabricated an array of 32 × 32 Au/Al hybrid-gate 2T0C DRAM cells and demonstrated its excellent performance in image storage. The operation process for image storage and readout is shown in Fig. 5a, with the circuit diagram of the 2T0C DRAM array presented in Fig. 5b. The original image is first converted to grayscale to obtain a 32 × 32 pixel matrix with 3-bit grayscale values, which is then mapped into a 3-bit voltage value matrix. The voltage values within the matrix are written into the 32 × 32 Au/Al gate 2T0C DRAM circuits. After holding for a period, a voltage pulse is applied to RWL, and the current values are sampled. The *V*_SN_ values on the storage nodes are obtained by allometric fitting of the *I−V* curves. The readout *V*_SN_ is then processed through a 3-bit ADC and mapped back to grayscale values, completing the retrieval of the image data stored in the 2T0C DRAM.

We observed that the readout image exhibits some distortion compared to the original image, which can be attributed to device noise in the write and readout systems and leakage current in the 2T0C units causing *V*_SN_ drop. Due to the high linearity of the write voltage-readout current mapping in the Au/Al hybrid-gate 2T0C, the distortion is significantly reduced compared to the Au/Au gate 2T0C structure (Fig. 5c). We then calculated the proportion of distorted pixel points and the similarity between the readout images and the original images after different storage durations (Fig. 5d). To evaluate the similarity between the readout and original images, we employed three parameters: the bit error rate (BER), normalized mean squared error (NMSE), and peak signal-to-noise ratio (PSNR) (see detailed calculation in Section S8) [31–34]. A smaller NMSE indicates a reduced deviation between the readout image pixels and the true pixels of the original image, while a larger PSNR value suggests better image quality. From the statistical results, it is evident that the readout accuracy of the Au/Al gate 2T0C DRAM circuits significantly outperforms that of the Au/Au gate 2T0C DRAM. After a retention time of 600 s, the BER of the hybrid-gate 2T0C DRAM remains below 5%, with an NMSE value of <0.008 and a PSNR value >30 dB, indicating low-distortion image readout.

## CONCLUSION

This study designs and fabricates a novel 2T0C DRAM structure based on monolayer MoS_2_ through the hybrid-gate process. By employing Au and Al gate electrodes for the *T*_write_ and *T*_read_, respectively, a stable and controllable negative shift in the *V*_TH_ of *T*_read_ is achieved, effectively addressing the contraction of the sensing window issue inherent in conventional 2T0C DRAMs. This enhances the sensing margin and enables high-precision, linear read/write operations. The device demonstrates a retention time exceeding 100 s, an off-state leakage current as low as 7 × 10^−17^ A/μm, and a write speed of 25 ns, while supporting distinguishable 3-bit data storage. Additionally, this work demonstrates the write, store, and read operations for a 32 × 32-pixel image. After 600 s of storage, the Au/Al gate 2T0C DRAM still maintains a bit error rate below 5%, enabling low-distortion image retrieval.

The demonstrated MoS_2_ 2T0C DRAM unit using hetero-processes exhibits outstanding non-destructive read capability, long retention, and multi-level data storage, highlighting its potential for high-density, low-power memory applications. Future research will further focus on scaling down the device to sub-micron channel lengths, implementing vertical 3D stacking, and further optimizing interface engineering, aiming to enable ultra-compact, high-speed, and CMOS-compatible embedded memory systems.

## METHODS

### Synthesis of monolayer MoS_2_

A monolayer MoS_2_ film was synthesized by chemical vapor deposition (CVD) on a 2-inch sapphire. In a tube furnace, molybdenum trioxide (MoO_3_) powder was used as the Mo source, and sulfur powder served as the S source, with a quartz substrate coated with Al_2_O_3_ placed at a fixed position. The chamber was evacuated to <1 Pa and purged with Ar gas until the chamber pressure stabilized at 0.2 MPa. The substrate region was then heated to 700°C, and the sulfur source was maintained at 220°C for a 15-min growth process, yielding a monolayer MoS_2_ film [35].

### Characterization of monolayer MoS_2_

We conducted atomic force microscopy (AFM) scans to measure the step height of the MoS_2_ film and obtained cross-sectional images using transmission electron microscopy (TEM). The results indicate a thickness of 0.75 nm (Fig. S1), consistent with monolayer MoS_2_. Raman and photoluminescence (PL) spectroscopy were performed at 15 random locations on a 2-inch MoS_2_ wafer (Fig. S2). The Raman spectrum exhibits an E^1^_2_ _g_ mode at 386 cm^−1^ and an A_1_ _g_ mode at 406 cm^−1^, which are consistent with reported monolayer MoS₂ values [36]. The PL spectrum shows a characteristic peak at ∼1.88 eV, corresponding to the bandgap of monolayer MoS_2_ [37]. The high consistency of the spectroscopic results confirms the uniformity of the MoS_2_ film.

### Fabrication of MoS_2_-based 2T0C DRAM

Figure 1e illustrates the fabrication flow of the 2T0C DRAM device. First, laser direct writing was used to define the source and drain regions on monolayer MoS_2_ film, followed by the deposition of a 35 nm Au layer using electron beam evaporation (EBE). Subsequently, reactive ion etching (RIE) was employed to pattern the MoS_2_ channel region. Next, a seeding layer was deposited via EBE. A HfO_2_ dielectric layer was deposited using atomic layer deposition (ALD). The top-gate regions were defined via EBE, with 35 nm Au deposited for *T*_write_ and a 5 nm Al/30 nm Au stack deposited for *T*_read_. Then via-hole regions were patterned on the source pad of *T*_write_, followed by etching using inductively coupled plasma (ICP), and then filled with Au. Finally, 35 nm Au was deposited via EBE to form metal interconnects between the source of *T*_write_ and the top gate of *T*_read_.

### Electrical measurement of 2T0C DRAM

The testing program is set using the B1500A semiconductor analyzer to apply voltages to the transistor for measuring the output and transfer characteristics (Fig. S3, Fig. S4). The *C*−*V* characteristics are measured using the E4990A impedance analyzer. The 81110A pulse generator applies voltage pulses to the source and gate of *T*_write_ and the B1500A semiconductor analyzer applies a voltage to the drain of *T*_read_, followed by measuring the *I*_DS_ variation to determine the voltage at the storage node and the retention characteristics (see detailed data in Section S5). All tests were conducted under ambient laboratory conditions (room temperature, atmospheric environment). Additional retention characteristics at elevated temperatures (85°C, 12 h) are shown in Section S11.

## Supplementary Material

nwaf555_Supplemental_File
