# Supplementary material for: Hybrid-gate MoS2 2T0C DRAM for low-power multi-bit storage with high linearity
Source: Natl Sci Rev. 2025 Dec 5;13(2):nwaf555. doi: 10.1093/nsr/nwaf555 (PMC12857206; doi:10.1093/nsr/nwaf555)
Supplement: nwaf555_Supplemental_File [file nwaf555_supplemental_file.pdf]

Supplementary Information

Hybrid-Gate MoS_2 2T0C DRAM for Low-Power Multi-Bit Storage with High Linearity

Zhejia Zhang[1,†], Saifei Gou[1,†], Yufei Song[1,†], Xiangqi Dong[1], Yuxuan Zhu[1], Zhengjie Sun[1], Mingrui Ao[1], Qicheng Sun[1], Jinshu Zhang[1], Yan Hu[1], Yuchen Tian[1], Haojie Chen[1], Xinliu He[1], Jieya Shang[1], Qihao Chen[1], Yang Liu[1], Yin Xia[2], Chen Yang[3], Hao Meng[3], Mingyuan Liu[3], Huihui Li[3], Yin Wang[2,*], Peng Zhou[1,2,*] and Wenzhong Bao[1,2,4,*]

[1]State Key Laboratory of Integrated Chip and Systems, School of Microelectronics, Fudan University, Shanghai 200433, China;

[2]Shaoxin Laboratory, Shaoxing 312000, China;

[3]Changxin Memory Technologies, Hefei 230601, China;

[4]Shanghai AtomIC Technology, Shanghai 201318, China

[*]**Corresponding authors**. E-mails: baowz@fudan.edu.cn; pengzhou@fudan.edu.cn; 18112020010@fudan.edu.cn

[†]Equally contributed to this work.

Section 1. Monolayer MoS_2 Characterization

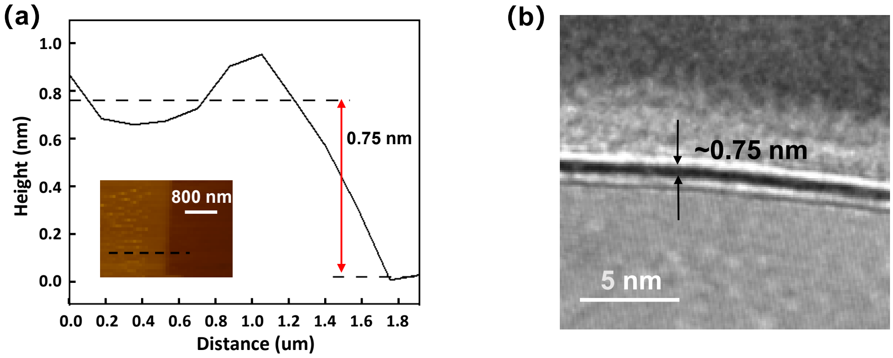

Figure S1 | AFM and TEM images of monolayer MoS_2. a, AFM image of the monolayer MoS_2, showing height difference data. b, Cross-sectional TEM image of the monolayer MoS_2. Both the AFM measurement and TEM analysis confirm that the thickness of the MoS_2 thin film is approximately 0.75 nm, consistent with the atomic thickness of monolayer MoS_2.

Section 2. Characterization of monolayer MoS_2

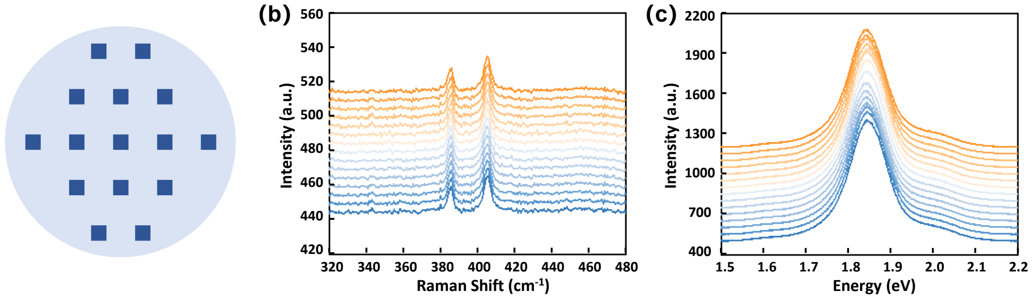

Figure S2 | The Raman and PL spectrums of 2 inch monolayer MoS_2 wafer. a, Schematic illustration of 15 characterization regions selected on a 2-inch MoS_2 wafer. b, Raman spectroscopy data from 15 measurement regions. c, PL spectroscopy data from 15 measurement regions. The Raman spectra exhibit the E_{2g}^{1} mode at 386 cm^{-1} and the A_{1g} mode at 406 cm^{-1}, while the PL spectra reveal a characteristic peak at approximately 1.88 eV, all of which are consistent with the optical signatures of monolayer MoS_2. Furthermore, the high consistency between the Raman and PL characterization results indicates the uniformity of the MoS_2 thin film over a large area.

Section 3. Transfer characteristics

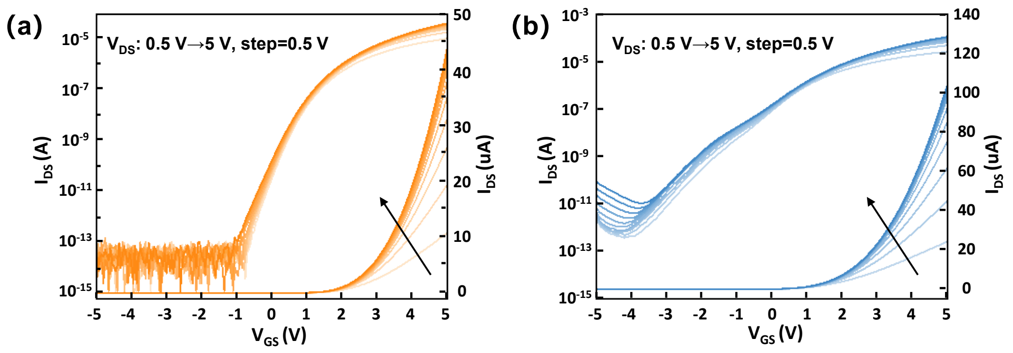

Figure S3 | The transfer characteristics of Au gate transistor and Al gate transistor. a, Transfer characteristic curves of MoS_2 transistors with Au gate. b, Transfer characteristic curves of MoS_2 transistors with Al gate. (V_{GS}: -5 V→5 V, V_{DS}: 0.5 V→5 V, step=0.5 V)

Section 4. Output characteristics

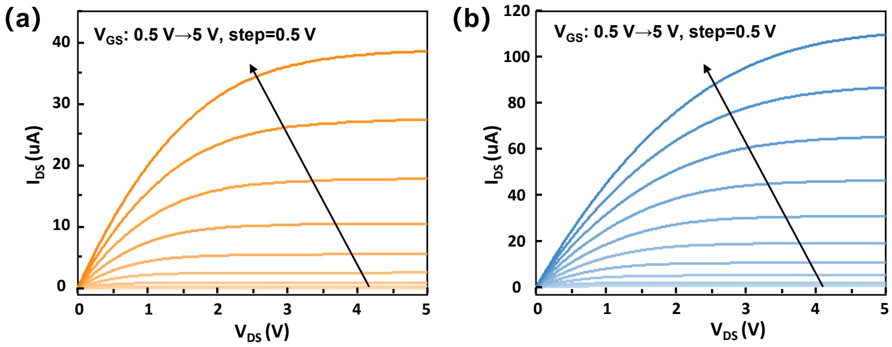

Figure S4 | The output characteristics of Au gate transistor and Al gate transistor. a, Output characteristic curves of MoS_2 transistors with Au gate. b, Output characteristic curves of MoS_2 transistors with Al gate. (V_{DS}: 0 V→5 V, V_{GS}: 0.5 V→5 V, step=0.5 V)

Section 5. Electrical measurement system

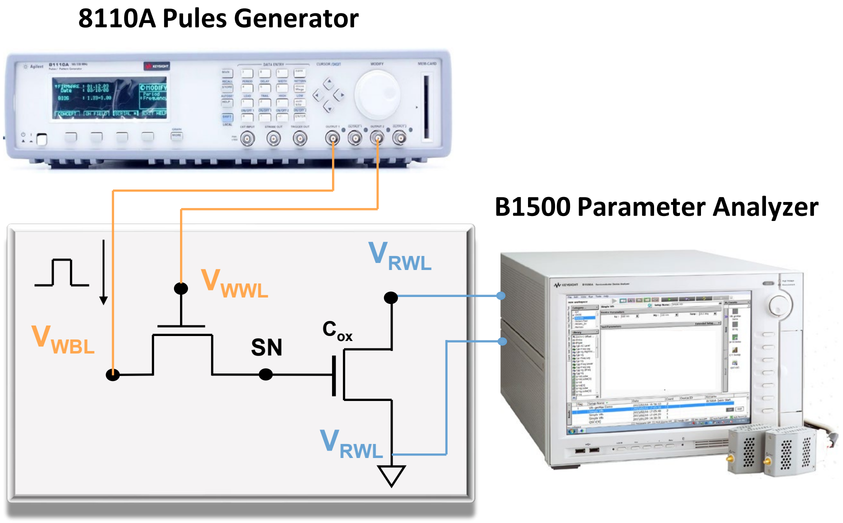

Figure S5 | Schematic diagram of the electrical measurement system. The diagram illustrates the electrical measurement process for evaluating the memory performance of the 2T0C device. A multi-channel pulse signal generator is used to generate write pulses, enabling the write operation. A semiconductor parameter analyzer measures the rWL current and monitors its temporal evolution, thereby obtaining the voltage at the SN node and assessing its retention characteristics.

Section 6. Retention characteristics

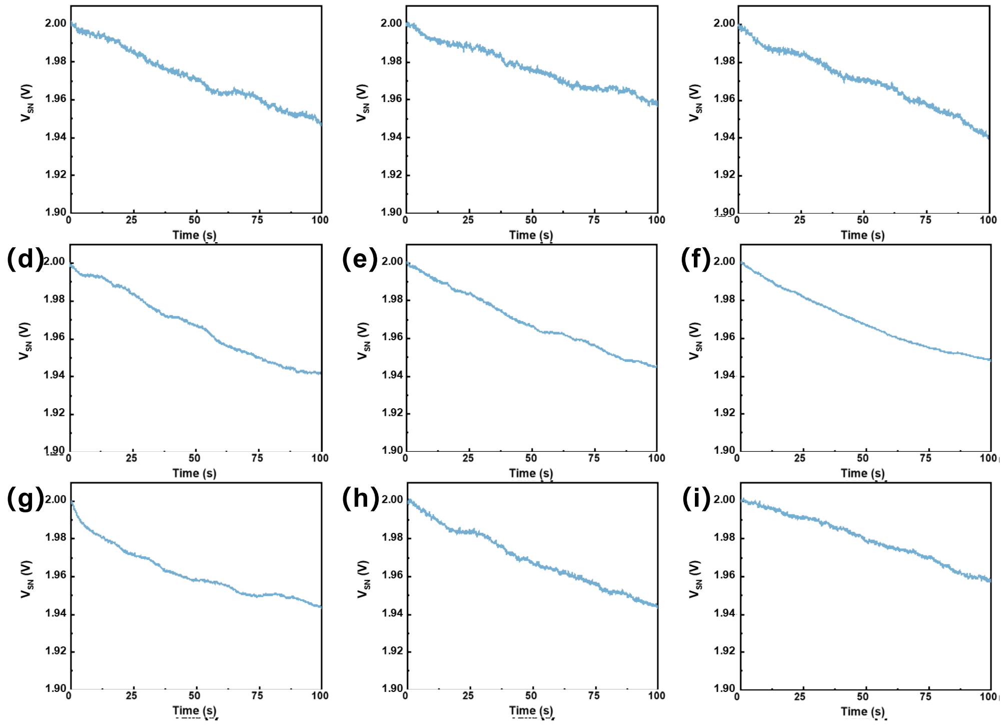

Figure S6 | a–i, Retention characteristic test data of nine Au/Al gate 2T0C cells. After a retention period of 100 s, all nine datasets exhibit a voltage drop of less than 0.1 V, demonstrating the excellent uniformity of the retention characteristics in Au/Al gate 2T0C cells.

Section 7. Energy consumption calculation

$$\boldsymbol{Energy\ consumption = \int V_{wBL} \cdot I(t)dt} \quad (1)$$

$$\boldsymbol{= V_{wBL} \int I(t)dt} \quad (2)$$

$$\boldsymbol{= V_{wBL} \cdot Q} \quad (3)$$

$$\boldsymbol{= V_{wBL} \cdot C_{ox} \cdot V_{SN}} \quad (4)$$

The lowest rBL value without threshold loss was selected as the reference for calculating the write energy consumption. For Au/Au gate 2T0C cells, wBL=1 V and V_{SN}≈0.9 V, while for Au/Al gate 2T0C cells, wBL=0.2 V and VSN≈0.14 V. The oxide capacitance is C_{ox}=1.5 pF.

$$\boldsymbol{\frac{Energy\ consumption(Au{-}Au\ 2T0C)}{Energy\ consumption(Au{-}Al\ 2T0C)} \approx 30} \quad (5)$$

Therefore, the Au-Al 2T0C cell structure can significantly reduce the write energy consumption by more than one order of magnitude.

Section 8. Accuracy calculation

$$\boldsymbol{MSE} = \frac{1}{mn}\sum_{i=0}^{m-1}\sum_{j=0}^{n-1}\left[I(i, j) - \hat{I}(i,j)\right]^2 \quad (1)$$

$$\boldsymbol{NMSE} = \frac{\sum_{i=0}^{m-1}\sum_{j=0}^{n-1}\left[I(i, j) - \hat{I}(i,j)\right]^2}{\sum_{i=0}^{m-1}\sum_{j=0}^{n-1}[I(i, j)]^2} \quad (2)$$

$$\boldsymbol{PSNR} = -10\, log_{10}\left(\frac{MSE}{MAX_I^2}\right) \quad (3)$$

I(i,j) denotes the pixel value of the retrieved image after storage, and $\hat{I}(i,j)$ represents the original pixel value. The parameters m and n correspond to the height and width of the image, respectively, while MAX_I denotes the maximum pixel value in the retrieved image.

The mean squared error (MSE), normalized mean squared error (NMSE), and peak signal-to-noise ratio (PSNR) are used as evaluation metrics. NMSE is an indicator of model prediction error, which normalizes the MSE to eliminate the effects of dimensionality and scale. PSNR, defined based on MSE, represents the ratio between the maximum possible signal power and the power of the corrupting noise. Both NMSE and PSNR are widely used for image quality assessment. In this study, NMSE and PSNR are employed to quantify the discrepancy between the stored and retrieved images and the original images.

Section 9. The endurance characteristics of the transistor

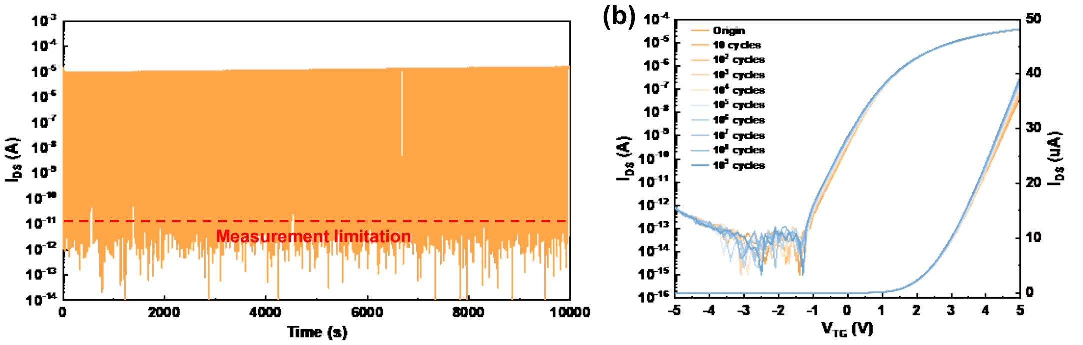

Figure S9 | The endurance characteristics of the transistor. a, Continuous I_{DS} testing under -3 V to 3 V V_{GS} bias scanning. b, Transfer curves of the transistor after different scanning cycles.

At the transistor level, we continuously applied a cyclic gate voltage sweeping from -3 V to 3 V (with V_{DS} = 1 V). Figure S9(a) shows the real-time variation of the transistor's source-drain current in response to the gate voltage changes. Furthermore, we measured the transfer characteristics of the transistor after different numbers of sweeping cycles, as presented in Figure S9(b). The experimental results indicate that even after more than 10^9 cycles, the transistor maintains excellent electrical performance without any observable degradation.

Section 10. The endurance characteristics of the 2T0C device

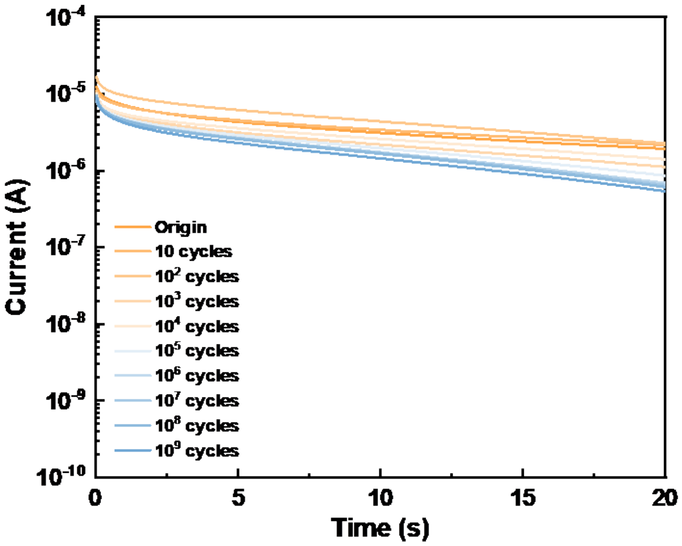

Figure S10 | Retention characteristics of the devices after undergoing different numbers of writing/erasing cycles.

For the 2T0C memory cell, we have evaluated its retention characteristics after different numbers of write/erase cycles, with the results presented in Figure S10. Comparative analysis clearly shows that the 2T0C cell developed in this work maintains its quasi-nonvolatile retention properties even after more than 10^9 write/erase cycles. This robust endurance performance further confirms the cell's suitability for practical DRAM applications.

Section 11. The retention characteristics at elevated temperatures

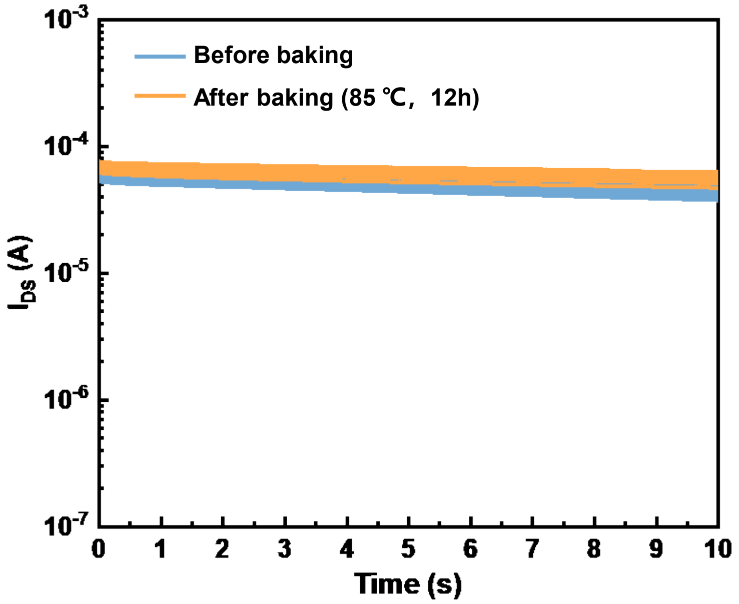

Figure S11 | Retention characteristics before and after baking (85°C, 12hours).

We measured the retention characteristics of 10 devices, subjected them to 85°C baking under atmospheric pressure for 12 hours to accelerate device aging, and then re-evaluated their retention performance. The results, presented in Figure S11, demonstrate that the retention characteristics remain largely consistent before and after the baking process, with no significant degradation observed. This confirms the robust reliability of the proposed 2T0C memory cells for real-world applications.

Section 12. Long-term stability of Al gate transistors

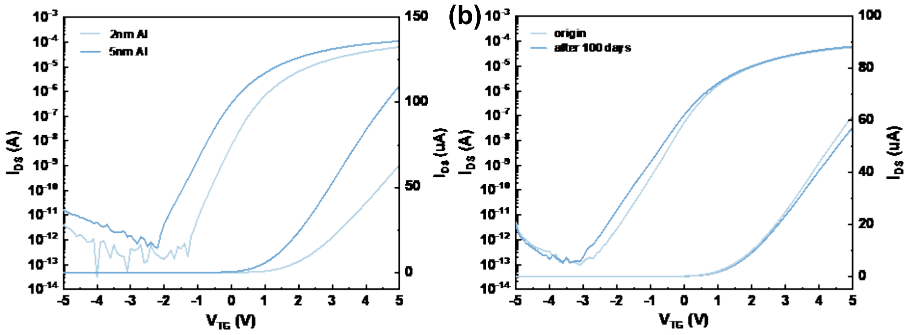

Figure S12 | Long-term stability of Al gate transistors. a, Comparison of transfer characteristics of transistors with different Al thickness. b, Comparison of transfer characteristics of Al gate transistors before and after 100 days.

Regarding the determination of the aluminum thickness, we conducted comparative experiments in our previous study, fabricating transistors with 2 nm and 5 nm Al as the work function modulation layer and evaluating their electrical performance (Figure S12(a)). We found that when the Al layer is too thin (2 nm), it becomes insufficiently dense and prone to oxidation, failing to provide effective threshold voltage modulation. Therefore, the 5 nm Al thickness was selected for optimal performance.

To further verify the long-term reliability of the Al gate transistors, we retrieved devices fabricated approximately 100 days ago and re-measured their electrical characteristics. As shown in Figure S12(b), the transfer characteristics remain virtually unchanged, with key device parameters such as threshold voltage, subthreshold swing, on-state current, and off-state current, showing negligible degradation. These results confirm the robust long-term stability of the 5 nm Al / 30 nm Au composite gate structure.

Section 13. Wafer-scale transistors fabrication

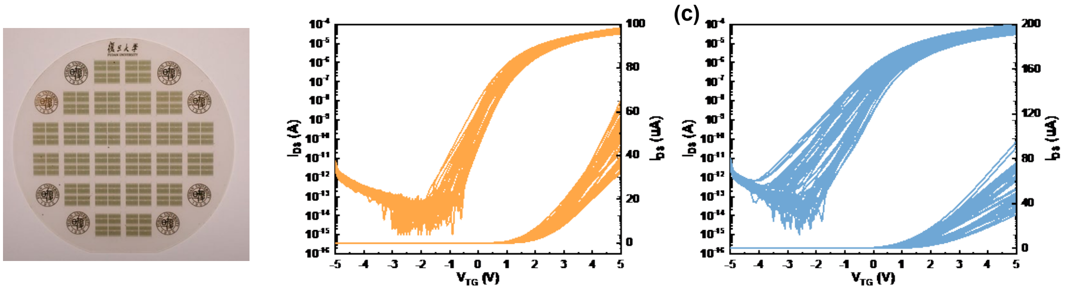

Figure S13 | Wafer-scale fabrication. a, Transistors and 2T0C cells array on a 4-inch wafer. b, Transfer characteristic curves of 50 randomly selected Au gate transistors. c, Transfer characteristic curves of 50 randomly selected Al gate transistors.

Section 14. Uniformity of Au/Al gate 2T0C devices

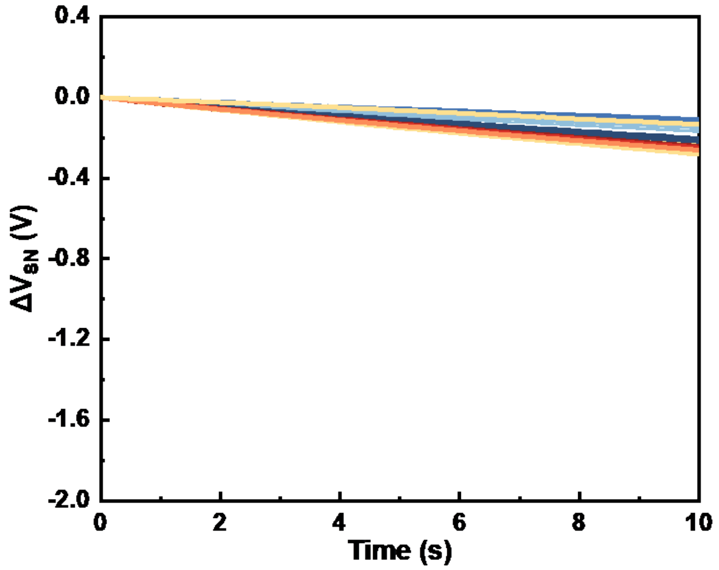

Figure S14 | Retention characteristic uniformity of 20 randomly selected Au/Al gate 2T0C devices.

Section 15. Leakage mechanisms

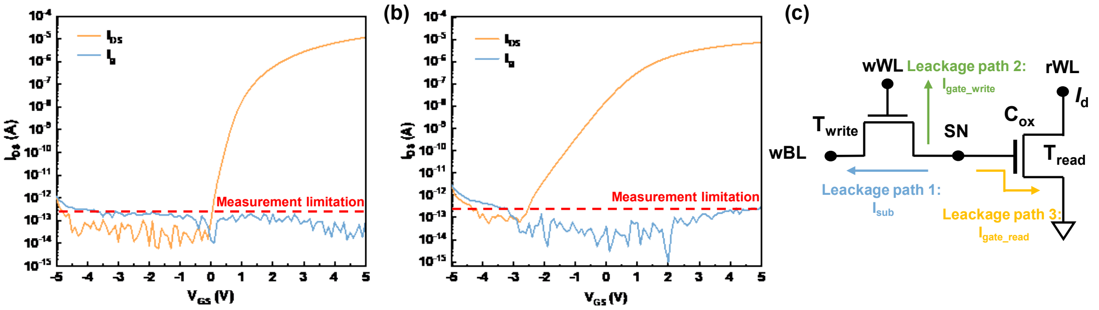

Figure S15 | Leakage mechanisms in the hybrid-gate 2T0C structure. a, Gate leakage measurement data of Au gate MoS_2 transistor. b, Gate leakage measurement data of Al-gate MoS_2 transistor. c, Three potential leakage paths in the 2T0C device.

As confirmed through discussions with ChangXin Memory Technologies (CXMT) and shown in Figure S15, the gate leakage currents of both the T_{write} and Tread are significantly lower than the off-state current of the T_{write}. Given this, these two gate leakage pathways are not the primary contributors to overall leakage and were therefore excluded from the leakage analysis conducted in this study.

Section 16. Allometric fitting

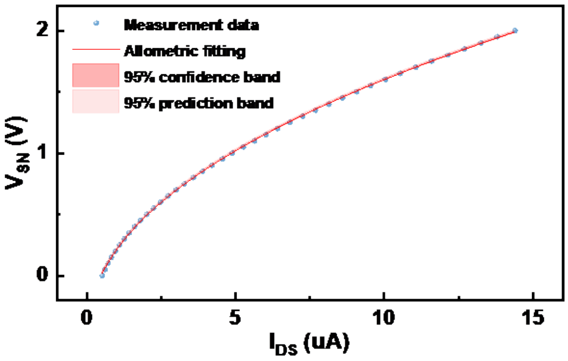

Figure S16 | Allometric fitting of V_{SN} versus I_{RBL} from experimental data.

Allometric: y = a + b*x^c	
**a**	**-0.52506 ± 0.01425**
**b**	**430.67317 ± 21.1743**
**c**	**0.46139 ± 0.00483**
**R^2**	**0.99986**

Table S1. Table of regression coefficients and R^2 for allometric fitting.

Since the potential variation of the storage node in the 2T0C device cannot be directly measured, we use the allometric model (y = a+b*x^c) to fit I_{DS} and V_{SN}. In order to ensure the accuracy of the fitting, the coefficient relationship of a, b and c must be adjusted so that the R^2 of the fitting curve tends to 1. As illustrated in Fig.S16, the values of a, b and c are set to a= -0.52506, b= 430.67317, c= 0.46139. The R^2 value for this set of values is 0.99986, which demonstrates the efficacy of the fitting method.

Section 17. Time-dependent reliability

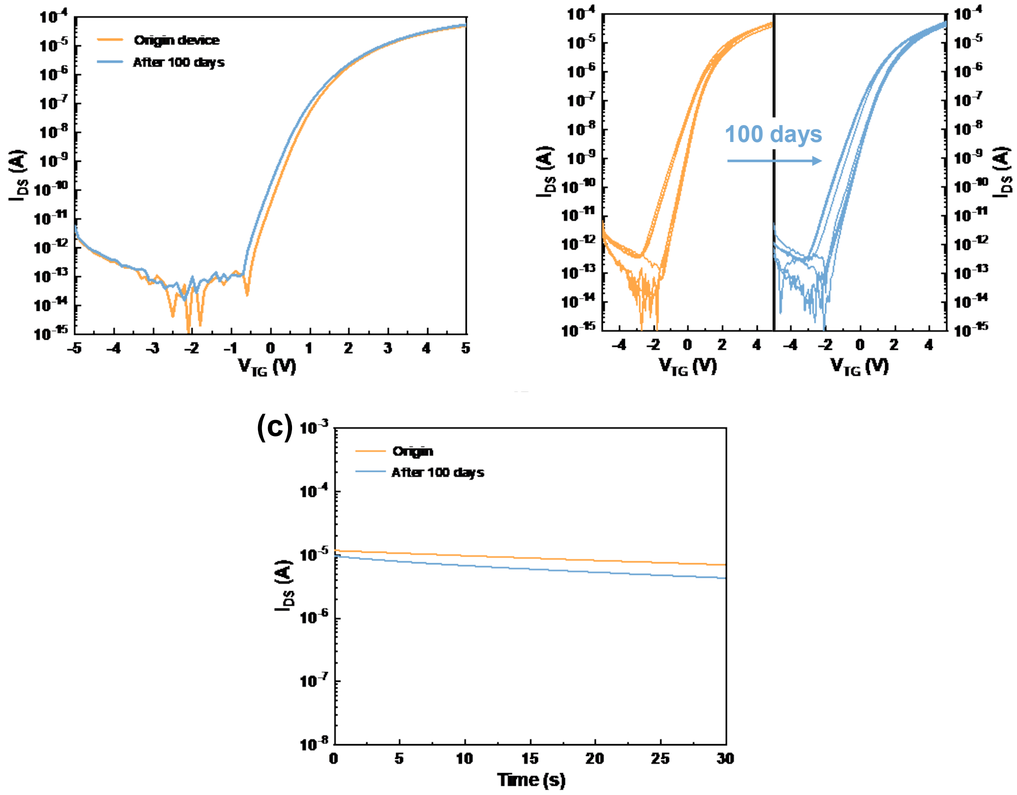

Figure S17 | The time-dependent reliability of the hybrid-gate 2T0C cell. a, Comparison of transfer characteristics of Au gate transistor before and after 100 days. b, Comparison of transfer characteristics of 10 Au gate transistors before and after 100 days. c, Comparison of retention characteristics of hybrid-gate 2T0C cell before and after 100 days.

We re-evaluated the electrical characteristics of devices fabricated 100 days earlier, comparing them with their original performance data. As shown in Figures S17(a)-(c), devices stored under ambient argon atmosphere exhibited no significant degradation in either the transfer characteristics of individual transistors or the memory performance of hybrid-gate 2T0C cells. These results provide clear evidence of excellent long-term data retention and temporal stability.
